# Enhanced glucose processing in gestational diabetes diagnosis: Effects on health equity and clinical outcomes

**DOI:** 10.1111/dme.15476

**Published:** 2024-12-17

**Authors:** Danielle L. Jones, Laura C. Kusinski, Peter Barker, Keith Burling, Ian Halsall, Elizabeth Turner, Coralie Glenn‐Sansum, Abby Rand, Jenny Finch, Genessa Peters, Geraldine Upson, Edward Mullins, Claire L. Meek

**Affiliations:** ^1^ Institute of Metabolic Science – Medical Research Laboratories, University of Cambridge Cambridge UK; ^2^ Leicester Diabetes Centre, University Hospitals Leicester & University of Leicester Leicester UK; ^3^ Cambridge Universities NHS Foundation Trust Cambridge UK; ^4^ Norfolk and Norwich University Hospitals NHS Foundation Trust Norwich UK; ^5^ North West Anglia NHS Foundation Trust Peterborough City Hospital Peterborough UK; ^6^ Salisbury NHS Foundation Trust Salisbury District Hospital Salisbury UK; ^7^ East Suffolk and North Essex Foundation Trust Ipswich Hospital, Ipswich Colchester UK; ^8^ Croydon Health Services NHS Trust Croydon University Hospital Croydon UK; ^9^ Imperial College London and the George Institute for Global Health London UK

**Keywords:** diagnosis, gestational diabetes, glucose analysis, HbA1c, oral glucose tolerance test, pre‐analytical factors, pregnancy

## Abstract

**Objectives:**

Gestational diabetes is diagnosed using an oral glucose tolerance test (OGTT), which has limited accuracy, reproducibility and practicality. We assessed the effect of enhanced pre‐analytical glucose processing upon glucose concentrations, gestational diabetes diagnosis, health equity and pregnancy outcomes, and if HbA1c was a suitable alternative.

**Methods:**

We recruited pregnant women with ≥1 risk factor to a prospective observational cohort study of pregnancy hyperglycaemia, endocrine causes, lipids, insulin and autoimmunity (OPHELIA), from nine UK centres. During a 75 g antenatal OGTT (National Institute of Health and Care Excellence criteria), standard glucose processing was compared to enhanced glucose processing (storage on ice, rapid centrifugation, aliquoting and freezing <2.5 h).

**Results:**

We recruited 1308 participants of mean (SD) age 31.5 years (5.0) and BMI 33.0 kg/m^2^ (6.8) of 82.5% white ethnicity, representative of the UK population. Enhanced glucose processing resulted in glucose levels ~0.6 mmol/L higher than standard glucose processing, increasing gestational diabetes diagnosis from 9% to 22%. Women with gestational diabetes on enhanced but not standard glucose processing (*n* = 165) were younger (31.9 vs. 33.2 years, *p* = 0.035), with a higher BMI (36.5 vs. 33.9 kg/m^2^; *p* = 0.003), different ethnic distribution (*p* = 0.025) and delivered more large‐for‐gestational age infants (37.0% vs. 22.3%; *p* = 0.006) compared to women with gestational diabetes on standard processing alone. HbA1c was not a suitable alternative predictor of gestational diabetes diagnosis (Area under receiver operator curve 0.74; 95% CI 0.68–0.79).

**Conclusions:**

An OGTT with enhanced glucose processing would support more accurate, equitable diagnosis of gestational diabetes, but with increased diagnosis rates.

## Graphical abstract

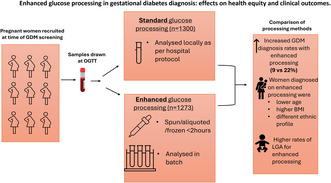

**What's new**

**What is already known?**

- Oral glucose tolerance tests are global standard for gestational diabetes diagnosis but are inaccessible and inconvenient for under‐represented groups.
- Enhanced methods of processing glucose samples may reveal undiagnosed cases, but its broader health equity impact remains unassessed.

**What has this study found/implications?**

- Enhanced glucose processing increased diagnosis from 9% to 22%.
- Women diagnosed with GDM using enhanced glucose processing methods, but not by standard glucose processing methods, had a higher BMI and different ethnicity profile and had more babies >90th centile (37%).
- HbA1c at 28 weeks' gestation was associated with LGA, but not other pregnancy outcomes including pre‐eclampsia, Caesarean delivery and preterm delivery.

## 1 INTRODUCTION

Gestational diabetes mellitus (GDM), the most common medical complication of pregnancy, is diagnosed using an oral glucose tolerance test (OGTT).^1^ The OGTT is known to have limited accuracy and reproducibility due to variations in pre‐analytical processing of glucose.^2^ Enhanced processing, including quicker centrifugation and processing of glucose, prevents degradation of glucose in the specimen tube yielding more accurate measurements of glucose. In clinical practice where standard processing pathways are used, centrifugation is often delayed resulting in loss of glucose in the specimen tube and falsely low concentrations. However, the effect of enhanced versus standard glucose processing upon GDM diagnosis rates, health equity and outcomes at a population level has not been assessed.

The National Institute for Health and Care Excellence (NICE) thresholds for the diagnosis of GDM on the OGTT (fasting glucose ≥5.6 mmol/L; 2‐h post‐load glucose ≥7.8 mmol/L)^3^ are based upon standard real‐world clinical data using standard processing methods, but associations between glucose concentrations and clinical outcomes using real‐world data are confounded by the treatment given. Estimates of risk using data from untreated women, such as during the Hyperglycaemia and Pregnancy Outcome (HAPO) study^4^ were also used to clarify associations between glucose thresholds and outcomes, which used enhanced methods of processing, common in clinical trial settings. When diagnostic thresholds are exceeded, there is an increased risk of adverse maternal–foetal outcomes including large‐for‐gestational age, neonatal hypoglycaemia and pre‐eclampsia.^4^

While the OGTT is standard for diagnosing GDM, women from socio‐economically deprived groups, Black ethnicity or with mental health issues are less likely to attend or complete OGTT appointments.^5^ Patient intolerability and poor reproducibility due to variable pre‐analytical sampling procedures also reduces validity in clinical practice.^6^ Delays in specimen processing results in metabolism of glucose by blood cells, reducing the plasma glucose concentration and complete inhibition of glucose metabolism in standard fluoride/EDTA tubes can be delayed for up to 4 h after sample collection.

Recent work has indicated that HbA1c could be an acceptable alternative to the OGTT for the diagnosis of GDM with suggested thresholds of 37 or 38 mmol/mol (5.5%–5.6%).^7^ While HbA1c is likely to have some linear associations with OGTT glucose measurements and GDM diagnosis, its ability to reliably predict suboptimal outcomes in women at risk of GDM is unclear.

The aim of the current study was to assess the effect of pre‐analytical processing of glucose upon glucose concentrations, GDM diagnosis, health equity and pregnancy outcomes in clinical practice. We also aimed to assess if HbA1c at 28 weeks' gestation was a suitable alternative diagnostic test in this population.

## 2 RESEARCH DESIGN AND METHODS

### 2.1 Study design and participants

We recruited women at risk of GDM to the Observational study for Pregnancy Hyperglycaemia, Endocrine causes, Lipids, Insulin and Autoimmunity (OPHELIA) study, a multisite prospective observational study aiming to identify underlying causes of GDM, between October 2018 and March 2023. Pregnant women with one or more risk factors^3^ were recruited between 24 and 28 weeks gestation prior to attending an OGTT as part of clinical testing for GDM. Women with singleton pregnancies and no severe congenital anomalies, and without confirmed diagnosis were eligible. Women were excluded if they had severe anaemia, pre‐existing diabetes prior to pregnancy or were taking medications at the time of the OGTT that may interfere with the results (e.g. corticosteroids). Nine study sites with a broad demographic representation participated, including Hinchingbrooke Hospital, Cambridgeshire; Queen Charlotte's and Chelsea Hospital, London; Lister, Hertfordshire; Colchester General Hospital, Essex; Norfolk and Norwich University Hospital, Norfolk; Peterborough City Hospital, Cambridgeshire; Salisbury District Hospital, Wiltshire; Croydon University Hospital, London; and Ipswich Hospital, Suffolk.

### 2.2 Ethical approval

Ethical approval for the study was granted by London‐Westminster Research Ethics Committee, London, United Kingdom (REC 18/LO/0477; research registry no. 5528). All procedures followed were in accordance with the ethical standards of the responsible committee on human experimentation, both institutional and national, in line the Declaration of Helsinki. All women provided written informed consent prior to study enrolment.

### 2.3 Data collection

Data were collected about medical and obstetric history, anthropometry, symptoms, ethnicity, family history of diabetes in first degree relatives, previous macrosomic infants (>4.5 kg), previous GDM and history of related conditions such as polycystic ovary syndrome. These data were unaffected by observer bias. Pregnancy outcome data were retrieved from the hospitals' electronic systems. GDM diagnosis was based upon the criteria of the United Kingdom outlined by the National Institute for Health and Care Excellence (NICE) (Fasting sample 0 min ≥5.6 mmol/L and/or 120 min ≥7.8 mmol/L).^3^

### 2.4 Biochemistry—standard processing

Pregnant women underwent a 2‐h OGTT following an overnight fast. Venous blood samples were obtained at 0 and 120 min after ingestion of a standard 75 g glucose drink. Additional blood samples were taken for HbA1c. Glucose and HbA1c were processed and analysed at local hospital laboratories in each study site using standard methods (hexokinase or glucose oxidase methods for glucose and Tosoh high performance liquid chromatography for HbA1c).

### 2.5 Biochemistry—enhanced processing

Samples of maternal plasma for glucose and insulin analysis were simultaneously collected in tubes containing lithium heparin at 0 min and 120 min, placed on ice prior to centrifugation, aliquoted and frozen at −20°C. All samples were processed within 2.5 h of the OGTT.

Glucose determination for the enhanced processing pathway used a Siemens Dimension hexokinase method, according to the manufacturers' guidelines (issue date 2019/04/01). Samples were defrosted and thoroughly mixed immediately prior to analysis. This assay has a working range of 0–27.8 mmol/L, a lower limit of detection of 0.056 mmol/L and high degrees of intra‐assay precision (CVs 1.2%–1.6% at concentrations ranges used in this study). Samples which were visibly lipaemic or haemolysed were not analysed. In our laboratory, inter‐assay precision was 2.5%–3.6% and internal quality control assessment was used immediately before and after running the samples in each batch.

### 2.6 Outcome definitions

Neonatal hypoglycaemia was defined as a plasma glucose level of less 2.6 mmol/L within the first 24 h of life. Neonatal Intensive Care Unit (NICU) admission included babies admitted for a period of at least 24 h within the first 28 days of life. Babies born before 37 weeks of gestation were considered preterm. LGA was calculated using intergrowth and GROW and defined as having a birthweight >90th centile.^8^, ^9^

### 2.7 Statistical analysis

Baseline characteristics, concentrations of glucose and insulin, HbA1c and pregnancy outcomes were presented as mean (SD) or *n* (%). We used Bland–Altman plots to assess the difference (bias) between glucose concentrations, at fasting and 2‐h post‐glucose load, using standard versus enhanced glucose processing in the overall cohort and by study centre. GDM diagnosis and pregnancy outcome rates were compared between groups (normal glucose tolerance, diagnosis using standard glucose processing, diagnosis using enhanced glucose processing but not standard glucose processing) using unadjusted logistic regression. We assessed the associations of HbA1c concentrations upon glucose concentrations at fasting and 2‐h post‐glucose load using unadjusted linear regression, and upon pregnancy outcomes using unadjusted logistic regression. The Area Under the Receiver Operator Characteristic curve (AUROC) was used to assess the ability of HbA1c to predict GDM diagnosis. Statistical analyses were performed using STATA software version 16.0 (Texas, USA) with significance threshold set at *p* < 0.05. Missing data were not imputed.

## 3 RESULTS

### 3.1 Differences between standard and enhanced glucose processing

**FIGURE 1:**
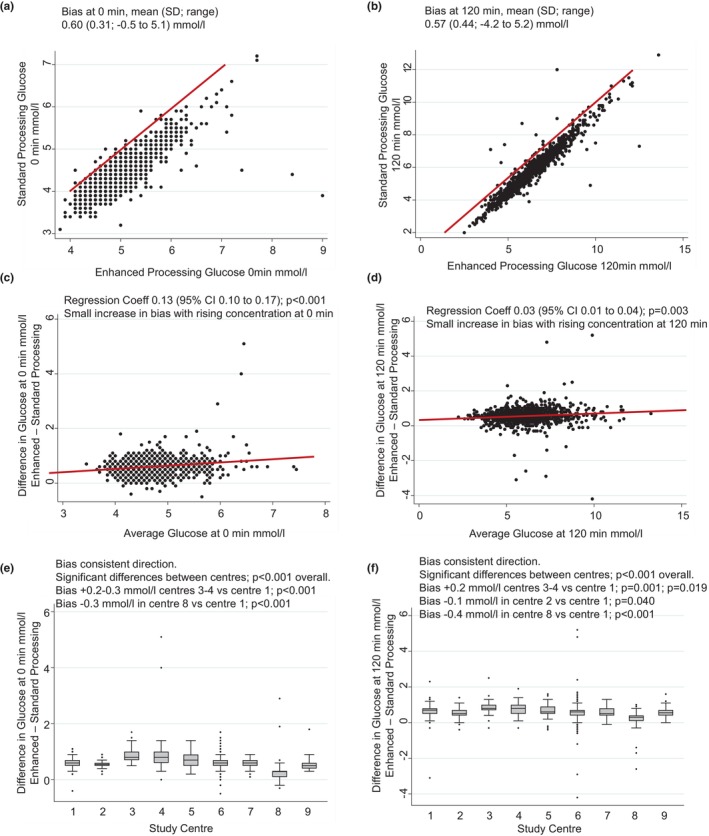
Enhanced processing of glucose was associated with glucose results of 0.6 mmol/L higher in fasting and 120‐min glucose concentrations (a, b), with a small increase in bias across the glucose concentration range (c, d). The bias was consistent in direction and magnitude in all nine study sites with small differences between individual study sites (e, f).

We recruited 1308 women with baseline characteristics described in Table 1. Enhanced processing of glucose was associated with glucose results of 0.60 mmol/L higher in fasting and 0.57 mmol/L higher in 120‐min post‐load glucose concentrations (Figure 1a,b). On Bland–Altman plots, the difference (bias) between standard and enhanced glucose concentrations showed a small increase with increasing concentration of glucose (Figure 1c,d). The difference between standard and enhanced glucose processing concentrations was of similar magnitude and direction across all nine study sites, with only small differences between individual study sites (Figure 1e,f; *p* < 0.001).

**TABLE 1:** Baseline characteristics and clinical outcomes of participants in the OPHELIA study presented as mean (SD) or *n* (%) where appropriate.

	n	All women *n* = 1308	1: NGT on both protocols *n* = 1022	2: GDM on standard protocol *n* = 121	3: GDM on enhanced protocol but not standard protocol *n* = 165	P 1 versus 2	P 1 versus 3	P 2 versus 3
Demographic details and reproductive history								
Maternal age years	1308	31.5 (5.0)	31.2 (4.9)	33.2 (4.7)	31.9 (5.4)	*p* < 0.001	*p* = 0.073	*p* = 0.035
BMI at study enrolment kg/m^2^	1249	33.0 (6.8)	32.3 (6.5)	33.9 (6.9)	36.5 (7.0)	*p* = 0.015	*p* < 0.001	*p* = 0.003
Gestational weight gain pre‐enrolment kg	1243	7.2 (6.0)	7.2 (5.5)	6.9 (4.7)	7.6 (9.3)	*p* = 0.588	*p* = 0.445	*p* = 0.458
Ethnicity %	White	1079/1308 (82.5%)	831/1022 (81.3%)	97/121 (80.2%)	151/165 (91.5%)	*p* = 0.025 across all groups		
	Asian	61/1308 (4.7%)	53/1022 (5.2%)	7/121 (5.8%)	1/165 (0.6%)			
	Black	127/1308 (9.7%)	101/1022 (9.9%)	14/121 (11.6%)	12/165 (7.3%)			
	Mixed/Other	41/1308 (3.1%)	37/1022 (3.6%)	3/121 (2.5%)	1/165 (0.6%)			
Multiparous	1308	790/1308 (60.4%)	613/1022 (60.0%)	73/121 (60.3%)	104/165 (63.0%)	*p* = 0.941	*p* = 0.457	*p* = 0.642
Previous GDM	1308	64/1308 (4.9%)	37/1022 (3.6%)	14/121 (11.6%)	13/165 (7.9%)	*p* < 0.001	*p* = 0.012	*p* = 0.264
First degree relative with T2D	1308	381/1308 (29.1%)	283/1022 (27.7%)	50/121 (41.3%)	48/165 (29.1%)	*p* = 0.002	*p* = 0.760	*p* = 0.031
Procedures at study visit								
Gestational age at OGTT	1289	28.2 (2.2)	28.2 (2.1)	28.0 (3.3)	28.3 (2.2)	*p* = 0.579	*p* = 0.440	*p* = 0.438
OGTT 0 h glucose mmol/L	1300	4.4 (0.5)	4.3 (0.3)	5.0 (0.7)	4.7 (0.5)	*p* < 0.001	*p* < 0.001	*p* < 0.001
OGTT 2 h glucose mmol/L	1296	5.8 (1.4)	5.4 (1.0)	8.5 (1.4)	6.6 (1.0)	*p* < 0.001	*p* < 0.001	*p* < 0.001
Enhanced processing 0 h glucose mmol/L	1273	5.0 (0.5)	4.8 (0.3)	5.5 (0.8)	5.5 (0.6)	*p* < 0.001	*p* < 0.001	*p* = 0.688
Enhanced processing 2 h glucose mmol/L	1250	6.4 (1.4)	5.9 (1.0)	8.9 (1.5)	7.4 (1.2)	*p* < 0.001	*p* < 0.001	*p* < 0.001
HbA1c mmol/mol	1236	31.7 (3.6)	31.0 (3.1)	34.8 (4.5)	33.2 (3.8)	*p* < 0.001	*p* < 0.001	*p* = 0.002
HbA1c %	1236	5.1 (2.5)	5.0 (2.4)	5.3 (2.6)	5.2 (2.5)	*p* < 0.001	*p* < 0.001	*p* = 0.002
Medication for Treating GDM								
On no medication for GDM	1299	1168/1299 (89.9%)	987/1022 (96.6%)	29/121 (24.0%)	152/165 (92.1%)	*p* < 0.001	*p* = 0.002	*p* < 0.001
Taking Metformin	1299	45/1299 (3.5%)	12/1022 (1.2%)	30/121 (24.8%)	3/165 (1.8%)	*p* < 0.001	*p* = 0.494	*p* < 0.001
Taking short‐acting insulin	1299	11/1299 (0.8%)	2/1022 (0.2%)	8/121 (6.6%)	1/165 (0.6%)	*p* < 0.001	*p* = 0.331	*p* = 0.004
Taking long‐acting insulin	1299	17/1299 (1.3%)	2/1022 (0.2%)	13/121 (10.7%)	2/165 (1.2%)	*p* < 0.001	*p* = 0.037	*p* < 0.001
Taking other insulin combinations	1299	15/1299 (1.2%)	1/1022 (0.1%)	11/121 (9.1%)	3/165 (1.8%)	*p* < 0.001	*p* < 0.001	*p* = 0.005
Outcomes								
Pre‐eclampsia	1280	16/1280 (1.3%)	9/1022 (0.9%)	3/121 (2.5%)	4/165 (2.4%)	*p* = 0.108	*p* = 0.079	*p* = 0.987
Gestational age at birth weeks	1284	39.3 (1.7)	39.4 (1.7)	38.3 (1.5)	39.3 (1.6)	*p* < 0.001	*p* = 0.402	*p* < 0.001
Preterm delivery	1216	68/1216 (5.6%)	47/1022 (4.6%)	11/121 (9.1%)	10/165 (6.1%)	*p* = 0.036	*p* = 0.417	*p* = 0.344
SVD	1282	666/1282 (52.0%)	537/1022 (52.5%)	58/121 (47.9%)	71/165 (43.0%)	*p* = 0.337	*p* = 0.023	*p* = 0.410
Caesarean delivery	1282	468/1282 (36.5%)	352/1022 (34.4%)	48/121 (39.7%)	68/165 (41.2%)	*p* = 0.254	*p* = 0.092	*p* = 0.793
Neonatal sex (men)	1275	646/1275 (50.7%)	504/1022 (49.3%)	65/121 (53.7%)	77/165 (46.7%)	*p* = 0.474	*p* = 0.498	*p* = 0.293
Birthweight INTERGROWTH centile	1272	65.1 (27.2)	63.8 (27.3)	65.8 (25.9)	72.1 (27.0)	*p* = 0.440	*p* < 0.001	*p* = 0.051
Birthweight GROW centile	1280	47.1 (29.6)	45.5 (29.0)	49.6 (29.7)	55.6 (32.0)	*p* = 0.139	*p* < 0.001	*p* = 0.110
LGA INTERGROWTH	1272	307/1272 (24.1%)	219/1022 (21.4%)	27/121 (22.3%)	61/165 (37.0%)	*p* = 0.920	*p* < 0.001	*p* = 0.006
LGA GROW	1280	140/1280 (10.9%)	87/1022 (8.5%)	15/121 (12.4%)	38/165 (23.0%)	*p* = 0.174	*p* < 0.001	*p* = 0.020
PPH	1280	441/1280 (34.5%)	329/1022 (32.2%)	51/121 (42.1%)	61/165 (37.0%)	*p* = 0.037	*p* = 0.241	*p* = 0.411
Neonatal hypoglycaemia	1280	28/1280 (2.2%)	17/1022 (1.7%)	9/121 (7.4%)	2/165 (1.2%)	*p* < 0.001	*p* = 0.663	*p* = 0.007
Neonatal Jaundice	1280	82/1280 (6.4%)	55/1022 (5.4%)	14/121 (11.6%)	13/165 (7.9%)	*p* = 0.008	*p* = 0.206	*p* = 0.304
NICU admission	1280	112/1280 (8.8%)	83/1022 (8.1%)	14/121 (11.6%)	15/165 (9.1%)	*p* = 0.218	*p* = 0.689	*p* = 0.511

*Notes:* Comparisons between groups were made using unadjusted linear or logistic regression. *p <* 0.05 indicates statistical significance.

Abbreviations: BMI, body mass index (kg/m^2^); GDM, gestational diabetes mellitus; LGA, large‐for‐gestational age; NICU admission, Neonatal Intensive Care Unit admission; OGTT, oral glucose tolerance test; PPH, postpartum haemorrhage; Preterm delivery, delivery <37 weeks gestation; SVD, spontaneous vaginal delivery.

### 3.2 Effect of enhanced glucose processing upon GDM diagnosis rates and treatment

**FIGURE 2:**
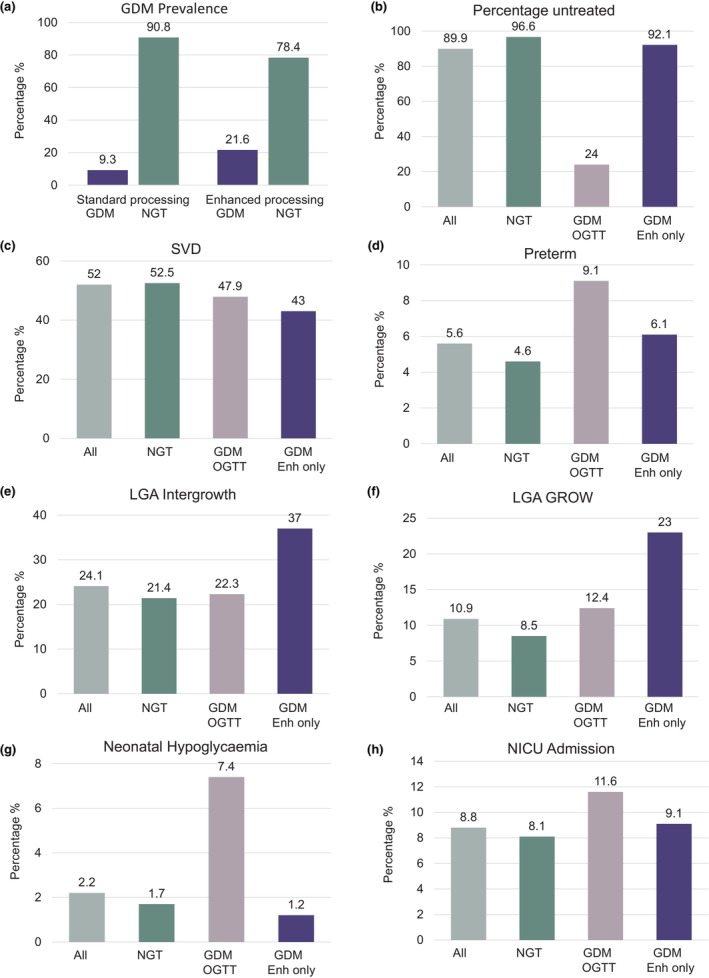
Differences between enhanced and standard glucose processing influenced gestational diabetes mellitus (GDM) diagnosis rates (a) and access to treatment (b). Women with GDM on enhanced processing only had fewer vaginal deliveries (c; *p* = 0.023) but comparable rates of preterm birth compared to women with normal glucose tolerance (NGT; d). Women with undiagnosed and untreated gestational diabetes had higher rates of LGA (e‐f; *p* < 0.001) but low rates of neonatal hypoglycaemia (although note ascertainment bias) and neonatal intensive care unit (NICU) admission (g, h).

Enhanced glucose processing resulted in 21.6% of women reaching diagnostic thresholds for GDM, compared to 9.3% on standard glucose processing (Figure 2a). The higher concentrations of glucose measured after the enhanced processing method were not explained by a bias in the measurement method (Figure SS1). In addition, 98% of women diagnosed using the standard protocol were also diagnosed on the enhanced glucose processing method. Among women with GDM diagnosed on standard glucose processing, 24% required medication to manage glucose levels at any stage in pregnancy compared to 8% in women who were diagnosed on enhanced glucose processing at 28 weeks gestation (92.1% on no medication vs. 24%; *p* < 0.001) (Figure 2b).

### 3.3 Missed diagnoses on standard versus enhanced glucose processing: Patient characteristics and health equity information

Using enhanced glucose processing, 165 (12.3%) women were diagnosed with GDM (Table 1). These women had a higher BMI compared to women with normal glucose tolerance (NGT) (36.5 (SD7.0) vs. 32.3 (6.5) kg/m^2^ NGT; *p* < 0.001) and women with GDM on standard glucose processing (36.5 (SD7.0) vs. 33.9 (6.9) kg/m^2^ GDM; *p* = 0.003), with similar amounts of weight gain in pregnancy prior to enrolment. Women with GDM on enhanced glucose processing were younger compared to women with GDM diagnosed on standard glucose processing (31.9 (5.4) vs. 33.2 (4.7) years; *p* = 0.035) and overall had a different ethnic distribution (*p* = 0.025), with a greater proportion of white women (91.5% vs. 80.2%).

### 3.4 Missed diagnoses on standard versus enhanced glucose processing: Glucose levels

Women diagnosed with GDM on enhanced glucose processing had comparable fasting glucose concentrations to women diagnosed on standard glucose processing (mean (SD) 5.5 mmol/L (0.6) vs. 5.5 mmol/L (0.8)), but lower 2‐h glucose concentrations (7.4 mmol/L (1.2) vs. 8.9 mmol/L (1.5); *p* < 0.001) (Table 1). Women with GDM on enhanced glucose processing only had HbA1c concentrations which were significantly higher than women with NGT (5.2% (2.5) vs. 5.0% (2.4)); (33.2 mmol/mol (3.8) vs. 31.0 mmol/mol (3.1); *p* < 0.001) but lower than women with GDM on standard glucose processing (5.2% (2.5) vs. 5.3% (2.6)); (33.2 mmol/mol (3.8) vs. 34.8 mmol/mol (4.5); *p* = 0.002) (Table 1).

### 3.5 Missed diagnoses on standard versus enhanced glucose processing: Differences in pregnancy outcomes compared to women with NGT

Women with GDM on enhanced glucose processing had significantly lower rates of vaginal deliveries (43% vs. 52.5%; *p* = 0.023), and significantly higher rates of LGA on both intergrowth (37% vs. 21.4% LGA; *p* < 0.001) and GROW centiles (23% vs. 8.5 LGA; *p* < 0.001) (Figure 2c,e,f and Table 1) compared to women with NGT. Rates of neonatal hypoglycaemia, admission to NICU and preterm delivery were similar to offspring of women with NGT (Figure 2d,g,h).

### 3.6 Missed diagnoses on standard versus enhanced glucose processing differences in pregnancy outcomes compared to women with diagnosed GDM

Women with GDM on enhanced glucose processing had significantly higher rates of LGA on both intergrowth (LGA 37% vs. 22.3%; *p* = 0.006) and GROW centiles (LGA 23% LGA vs. 12.4%; *p* = 0.02) compared to women with GDM on standard glucose processing (Figure 2e,f). Neonatal hypoglycaemia rates were lower in women with GDM on enhanced glucose processing (1.2% vs. 7.4%; *p* = 0.007) compared to women with GDM on standard glucose processing (Figure 2g); however, testing for hypoglycaemia is not routine unless neonates are identified as high risk. The rates of vaginal deliveries, admission to NICU and preterm delivery were similar between the groups (Figure 2c,d,h).

### 3.7 Predictive capability of HbA1c for GDM diagnosis and pregnancy outcomes

**FIGURE 3:**
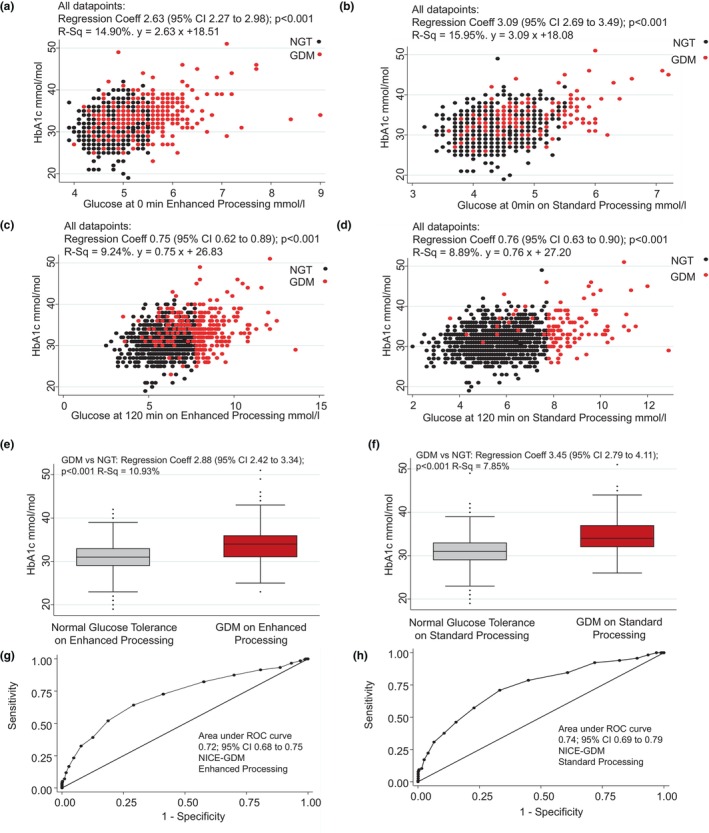
HbA1c was associated with fasting glucose on enhanced and standard processing (a, b) and 120‐min glucose (c, d). HbA1c was associated with gestational diabetes mellitus (GDM) diagnosis, but showed considerable degrees of overlap with women with normal glucose tolerance (NGT; e, f). Receiver operator curves (ROC) showed that HbA1c had modest ability to predict GDM diagnosis (g, h).

HbA1c showed a wide spread of values in association with fasting glucose (Figure 3a,b) and 120‐min glucose (Figure 3c,d) when measured using enhanced or standard glucose processing. Overall, HbA1c was significantly higher in women with GDM (Figure 3e,f; *p* < 0.001), although there was considerable overlap with levels in women with NGT. HbA1c was able to predict GDM diagnosis using enhanced glucose processing procedures (AUROC 0.72 95% CI: 0.68–0.75) and standard glucose procedures (AUROC 0.74 95% CI: 0.69–0.79) (Figure 3g,h). HbA1c was significantly associated with LGA (LGA Intergrowth; OR 1.05 (1.01–1.09; *p* = 0.012)); LGA GROW; OR 1.07 (1.02–1.12; *p* = 0.009) but not with other pregnancy outcomes across the cohort or by women diagnosed with GDM on enhanced glucose processing (Table S1).

## 4 DISCUSSION

Enhanced pre‐analytical procedures for glucose assessment during the antenatal OGTT increased GDM diagnosis rates (22% vs. 9%) but identified a group of women at high risk of LGA (37%) who were undiagnosed and untreated using standard testing procedures. Women with a missed diagnosis did not have consistently milder hyperglycaemia: They had comparable fasting glucose results but lower post‐load glucose and lower HbA1c compared to women who were diagnosed and treated. The likelihood of a missed diagnosis did not affect the population equitably in terms of age, BMI or ethnicity.

### 4.1 Strengths and weaknesses

The OPHELIA study is a large, multicentre study, including women with a wide range of BMI values and ethnic diversity representative of the United Kingdom overall. All study sites referred to the NICE guidelines for GDM diagnosis and treatment, and a protocol for enhanced glucose processing, providing consistency across the cohort.^3^ Despite the overall sample size, the number of women in groups categorised by ethnicity or less common outcomes such as neonatal hypoglycaemia are small, limiting the opportunity for subgroup analysis. Citrate tubes reduce pre‐analytical glucose loss in clinical practice, affecting GDM diagnosis rates.^10^ However, they were not included due to incompatibility with existing hospital phlebotomy equipment at the study sites when the study commenced. OPHELIA recruited throughout the Covid‐19 pandemic at study sites maintaining standard OGTT testing, ensuring consistent procedures despite pandemic‐related challenges in antenatal care.

### 4.2 Glucose measurement error: Relevance to other studies

We identified an increase of 12.3% in the rate of GDM diagnosis using an enhanced pre‐analytical procedure for glucose handling during the antenatal OGTT. Similar work by Potter and colleagues reported an increase in diagnosis rates of 9.0%.^11^ Munk Scheuer and colleagues identified that pre‐analytical factors accounted for a 11.1% difference in GDM prevalence between laboratories.^12^ However, Szoke and colleagues found larger differences and reported an increase in GDM diagnosis rate of 15.6% using citrate tubes.^10^

Recent work found biases in glucose measurement with varying pre‐analytical procedures of 0.19–0.30 mmol/L in Denmark,^12^ and 0.16–0.34 mmol/L in a larger study in Australia which increased GDM diagnosis rate from 11.6% to 20.6%.^11^ Furthermore, storing samples on ice prior to centrifugation increased diagnosis rates from 10.8% to 28.5%.^13^ However, it remains unclear if adapting pre‐analytical protocols influences pregnancy outcomes at a population level, as theoretically, only women with the mildest degrees of GDM would be undiagnosed.

### 4.3 Does improving diagnosis enhance pregnancy outcomes?

Our study identified that women with GDM on enhanced but not standard processing had a 37% risk of giving birth to an LGA infant. Since this was not an interventional study, we have no evidence that diagnosing and treating GDM in this group would have improved outcomes, reduced LGA or resulted in cost benefits to the health service. However, other work has shown that preventing LGA is one key outcome of treating GDM, even in cases considered to have mild hyperglycaemia only.^14^, ^15^ Data from the United Kingdom demonstrate that women with elevated fasting glucose levels consistent with GDM who are not diagnosed or treated have a four times increased risk of stillbirth.^16^ Given the higher rates of stillbirth in the United Kingdom compared to the rest of Europe, we consider that improving the accuracy of glucose testing in pregnancy may be a useful way of reducing perinatal mortality.

### 4.4 Does improving diagnosis enhance the risk of over‐treatment?

Song and colleagues reported 25.7% increase in GDM prevalence when citrate‐buffered tubes were used which they considered a profound over‐diagnosis.^17^ The authors disputed the thresholds recommended using data from the HAPO study^18^, ^19^ and noted that LGA rates of 10% occurred at different glucose thresholds for populations worldwide.^17^ However, these studies had a smaller sample size compared to the HAPO data, and were confounded by treatment provided during pregnancy. Additionally, women with mild GDM may be overtreated with birthweights below that of the healthy population.^20^, ^21^ This assumes that women identified have very mild degrees of hyperglycaemia and are at low risk of hyperglycaemia‐related sequalae. However, in our study, the degree of bias between standard and enhanced glucose results was variable and inconsistent throughout the population. Compared to women who were clinically diagnosed, women diagnosed on enhanced processing had comparable concentrations of fasting glucose and higher rates of LGA, suggesting that they did not have uniformly milder disease. Fasting hyperglycaemia is known to be strongly related to foetal growth and LGA^18^, ^22^ and is likely to be responsible for the high LGA rate.

There are other sources of biological variation affecting OGTT results which we have not assessed.^23^ The accuracy of the OGTT can be influenced by seasonal temperatures^24^ and carbohydrate intake in the day prior to testing.^25^ Additionally, individuals having two OGTTs within a week will only receive the same result on around 27%–80% of occasions.^26^, ^27^, ^28^ However, the impact of additional sources of variability on the OGTT results is likely to be randomly distributed across this cohort, thus the findings remain valid and of interest.

Our findings indicate HbA1c was not associated with GDM diagnosis or relevant pregnancy outcomes. Despite the advantages of HbA1c measurement such as its international standardisation and ability to be measured in a non‐fasted state, it is affected by altered red cell turnover, may not respond to short‐term hyperglycaemia in response to rising insulin resistance of pregnancy and may not perform consistently in states of low iron, folate or Vitamin B12.^29^, ^30^

### 4.5 Main implications for future work

Our work demonstrates that an OGTT with standard glucose processing and HbA1c are not able to accurately identify all cases of GDM. We focussed on specimen handling, but there are other pre‐analytical factors including diet, physical activity, poor sleep and stress which may also influence results.^2^ Concerningly, we identified that the inaccuracies in glucose processing do not equally affect women according to risk, but rather preferentially reduce diagnosis in women in the highest BMI groups who often have fasting hyperglycaemia. Future work should seek to understand why these inaccuracies disproportionately affect these groups and to identify a reproducible, accurate, equitable test for GDM.

## 5 CONCLUSION

Neither an oral glucose tolerance test with standard glucose pre‐analytical processing procedures, nor HbA1c offers consistently accurate diagnosis of GDM. Efforts to improve the accuracy of GDM diagnosis are warranted.

## AUTHOR CONTRIBUTIONS

Claire L. Meek was responsible for the conceptualisationconceptualization, design and methodology, data analysis and wrote and revised the written report. Danielle L. Jones had oversight of study coordination, database management, data analysis and writing the final report. Laura C. Kusinski assisted with study coordination, database management, data analysis and writing the final report. Elizabeth Turner, Coralie Glenn‐Sansum, Abby Rand, Jenny Finch, Genessa Peters, Geraldine Upson, and Edward Mullins were responsible for participant recruitment, data collection, and sample processing at their respective hospital sites. Peter Barker, Keith Burling and Ian Halsall were responsible for the laboratory assessment of the blood samples. Funding acquisition for this study was obtained by Claire L. Meek who is the guarantor for this work is, and as such, had full access to all the data in the study and takes responsibility for the integrity of the data and the accuracy of the data analysis. All authors reviewed the final version before submission. For the purpose of open access, the author has applied a Creative Commons Attribution (CC BY) licence to any Author Accepted Manuscript version arising from this submission.

## FUNDING INFORMATION

The OPHELIA study was funded by the EFSD‐Sanofi innovative outcomes project (2017; OPHELIA pilot study), NIHR Cambridge BRC and NIHR Leicester BRC. CLM is supported by Diabetes UK through an intermediate clinical fellowship (17/0005712; ISRCTN number 90795724) and the EFSD/Novo Nordisk Foundation Future Leader's Award (NNF19SA058974). The funders did not play a role in the design or conduct of the research study.

## CONFLICT OF INTEREST STATEMENT

None of the authors disclosed any financial or non‐financial competing interests in this study.

## Supporting information


**Figure S1.** Results from UK National External Quality Assurance (NEQAS) showing that there is no systematic bias in the Dimension method when compared to the national consensus values. The Dimension method used for enhanced processing in this study is coded as 15BE.

**Data S1.** Supporting Information.
