# Supplementary material for: Enhanced glucose processing in gestational diabetes diagnosis: Effects on health equity and clinical outcomes
Source: Diabet Med. 2024 Dec 17;42(3):e15476. doi: 10.1111/dme.15476 (PMC11823314; doi:10.1111/dme.15476)
Supplement: Supplementary file 1 — Figure S1. Results from UK National External Quality Assurance (NEQAS) showing that there is no systematic bias in the Dimension method when compared to the national consensus values. The Dimension method used for enhanced processing in this study is coded as 15BE. [file DME-42-e15476-s002.pptx]

## Slide 1
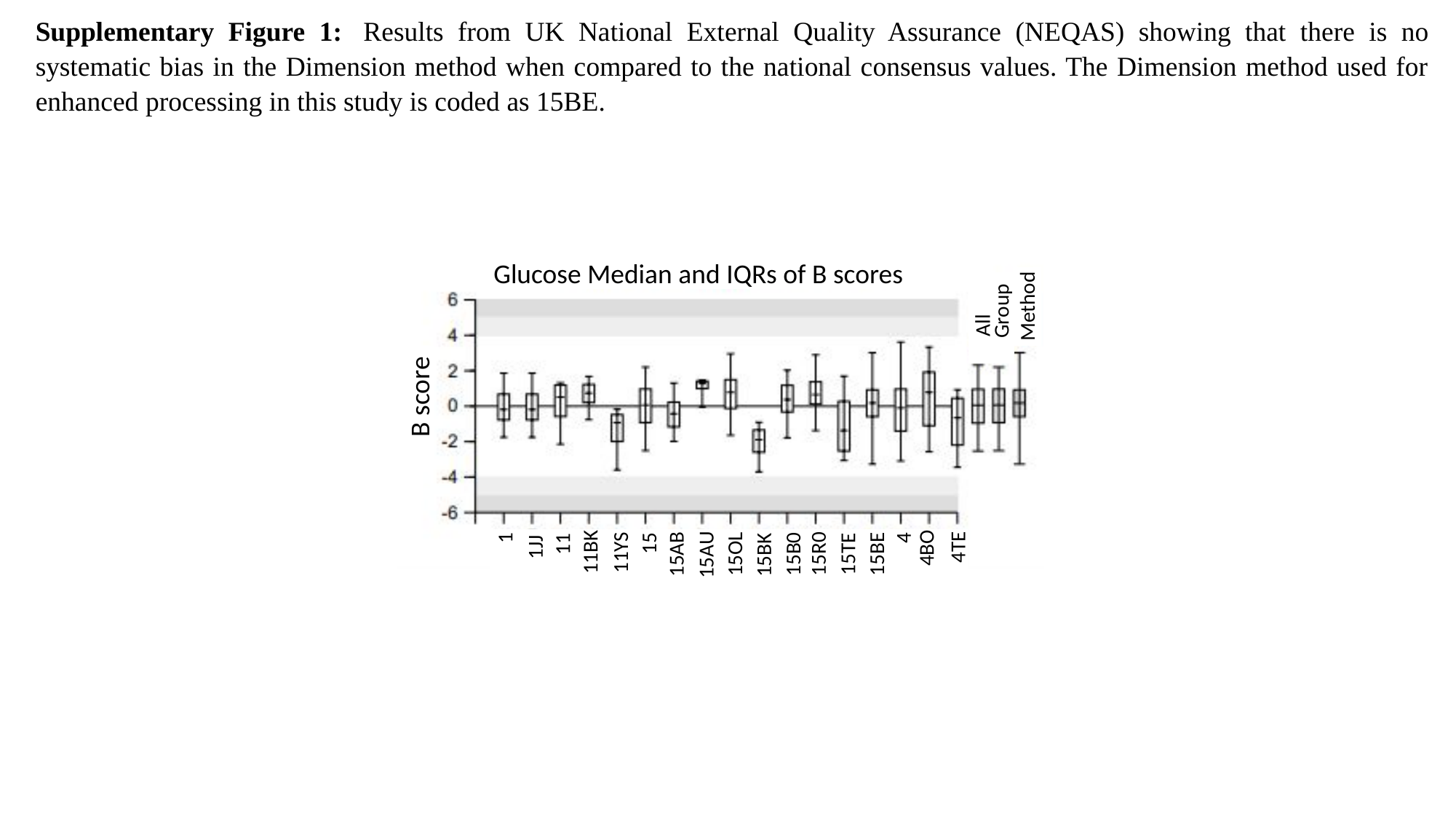

Supplementary Figure 1:  Results from UK National External Quality Assurance (NEQAS) showing that there is no systematic bias in the Dimension method when compared to the national consensus values. The Dimension method used for enhanced processing in this study is coded as 15BE.
Glucose Median and IQRs of B scores
B score
Method
Group
All
4
1
15
11
4TE
1JJ
4BO
11BK
11YS
15BE
15R0
15TE
15AB
15OL
15B0
15BK
15AU
