# Supplementary material for: Enhanced glucose processing in gestational diabetes diagnosis: Effects on health equity and clinical outcomes
Source: Diabet Med. 2024 Dec 17;42(3):e15476. doi: 10.1111/dme.15476 (PMC11823314; doi:10.1111/dme.15476)
Supplement: Supplementary file 2 — Data S1. Supporting Information. [file DME-42-e15476-s001.docx]

**Supplementary Table 1:** Associations between HbA1c, fasting glucose and 2-hr glucose on pregnancy outcomes. Significance testing is given for unadjusted logistic regression (OR (95% CI); p).

| Continuous measures of glucose | HbA1c  OR (95% CI); p | OGTT 0hr  OR (95% CI); p | OGTT 2hr  OR (95% CI); p |
| --- | --- | --- | --- |
|  |  | **Standard glucose processing** | **Standard glucose processing** |
| Pre-eclampsia | 1.12 (1.00 to 1.26); p=0.055 | 1.96 (1.02 to 3.76); p=0.043 | 1.21 (0.89 to 1.64); p=0.222 |
| Polyhydramnios | 1.02 (0.94 to 1.10); p=0.618 | 1.16 (0.72 to 1.88); p=0.533 | 1.03 (0.86 to 1.24); p=0.740 |
| Preterm delivery | 1.04 (0.97 to 1.11); p=0.257 | 1.18 (0.76 to 1.82); p=0.458 | 1.21 (1.03 to 1.41); p=0.018 |
| SVD delivery | 0.98 (0.95 to 1.01); p=0.192 | 0.80 (0.65 to 0.98); p=0.035 | 0.89 (0.82 to 0.96); p=0.003 |
| CS delivery | 1.02 (0.99 to 1.06); p=0.171 | 1.20 (0.97 to 1.48); p=0.099 | 1.06 (0.98 to 1.15); p=0.143 |
| Forceps | 1.00 (0.95 to 1.06); p=0.987 | 1.21 (0.85 to 1.73); p=0.298 | 1.18 (1.04 to 1.35); p=0.013 |
| Ventouse | 1.03 (0.95 to 1.12); p=0.460 | 1.19 (0.68 to 2.09); p=0.542 | 1.12 (0.91 to 1.39); p=0.285 |
| LGA Intergrowth | 1.05 (1.01 to 1.09); p=0.012 | 1.51 (1.20 to 1.91); p=0.001 | 1.14 (1.04 to 1.25); p=0.004 |
| LGA Grow | 1.07 (1.02 to 1.12); p=0.009 | 1.72 (1.28 to 2.29); p<0.001 | 1.29 (1.15 to 1.44); p<0.001 |
| PPH | 1.01 (0.98 to 1.04); p=0.505 | 1.23 (0.99 to 1.53); p=0.057 | 1.12 (1.03 to 1.21); p=0.008 |
| Neonatal hypoglycaemia | 1.07 (0.96 to 1.18); p=0.215 | 1.48 (0.82 to 2.70); p=0.196 | 1.49 (1.20 to 1.85); p<0.001 |
| Jaundice | 0.97 (0.91 to 1.04); p=0.356 | 1.09 (0.72 to 1.64); p=0.698 | 1.18 (1.02 to 1.36); p=0.031 |
| NICU admission | 1.01 (0.96 to 1.07); p=0.670 | 1.05 (0.73 to 1.52); p=0.775 | 1.01 (0.88 to 1.16); p=0.903 |
|  |  |  |  |
|  |  | **Enhanced glucose processing** | **Enhanced glucose processing** |
| Pre-eclampsia |  | 2.36 (1.06 to 5.23); p=0.035 | 1.22 (0.89 to 1.66); p=0.222 |
| Polyhydramnios |  | 1.74 (1.06 to 2.86); p=0.030 | 1.08 (0.90 to 1.30); p=0.399 |
| Preterm delivery |  | 1.07 (0.64 to 1.78); p=0.806 | 1.18 (1.01 to 1.39); p=0.042 |
| SVD delivery |  | 0.83 (0.65 to 1.04); p=0.109 | 0.90 (0.83 to 0.97); p=0.006 |
| CS delivery |  | 1.26 (0.99 to 1.60); p=0.061 | 1.06 (0.98 to 1.15); p=0.147 |
| Forceps |  | 1.05 (0.69 to 1.59); p=0.836 | 1.16 (1.01 to 1.32); p=0.031 |
| Ventouse |  | 0.97 (0.49 to 1.90); p=0.926 | 1.10 (0.90 to 1.36); p=0.353 |
| LGA Intergrowth |  | 1.52 (1.16 to 1.98); p=0.002 | 1.11 (1.02 to 1.22); p=0.020 |
| LGA Grow |  | 1.76 (1.26 to 2.48); p=0.001 | 1.22 (1.09 to 1.37); p=0.001 |
| PPH |  | 1.32 (1.03 to 1.68); p=0.027 | 1.13 (1.04 to 1.23); p=0.003 |
| Neonatal hypoglycaemia |  | 2.52 (1.37 to 4.63); p=0.003 | 1.51 (1.22 to 1.87); p<0.001 |
| Jaundice |  | 1.42 (0.92 to 2.21); p=0.114 | 1.19 (1.03 to 1.38); p=0.019 |
| NICU admission |  | 1.10 (0.73 to 1.65); p=0.646 | 0.98 (0.85 to 1.13); p=0.793 |
